# Robot-assisted laparoscopic varicocelectomy in a pediatric population

**DOI:** 10.1007/s00383-023-05488-w

**Published:** 2023-05-20

**Authors:** Susanne Reinhardt, Jorgen Thorup, Peter Hjorth Joergensen, Mikkel Fode

**Affiliations:** 1 Department of Pediatric Surgery, Rigshospitalet, Blegdamsvej 9, 2100 Copenhagen, Denmark; 2 Department of Clinical Medicine, Faculty of Health and Medical Sciences, University of Copenhagen, Blegdamsvej 3B, 2200 Copenhagen, Denmark; 3 Department of Urology, Copenhagen University Hospital, Herlev and Gentofte Hospital, Borgmester Ib Juuls Vej 1, 2730 Herlev, Denmark

**Keywords:** Pediatric surgery, Pediatric urology, Robotic surgery, Robotic urologic surgery, Varicocele, Varicocelectomy

## Abstract

**Purpose:**

To present our experience with robot-assisted laparoscopic varicocelectomy in a pediatric population.

**Methods:**

We reviewed 49 consecutive cases performed by the same experienced surgeon. One-to-four veins were ligated at the internal ring of the inguinal canal, while the testicular artery and lymphatics were spared. Information on patient characteristics, surgical time, complications, and recurrences were collected.

**Results:**

Median patient age was 14 (range 10–17) years. Forty-eight had left-sided varicoceles and one had a bilateral varicocele. Forty-five were grade 3. All patients were referred due to discomfort/pain and 20 also had reduced testicular size. The median operating time from skin incision was 48 min (31–89 min) and the median console time was 18 min (7–55 min). Forty-seven patients were discharged the same day. Two patients experienced pain and problems urinating, respectively. These issues had resolved by the first post-operative day. There were no other complications, but at 6 months, eight recurrences were noted (16%). Scrotal complaints had subsided in all patients. Catch-up growth of the affected testicles was seen in 19/20 cases.

**Conclusion:**

Robot-assisted laparoscopic varicocelectomy is feasible and safe in a pediatric population but with a relatively high recurrence rate.

## Introduction

A varicocele is defined a dilation of the veins in the pampiniform plexus within the scrotum. The condition usually arises in puberty with a prevalence of < 1% in boys aged 2–10 years, increasing to about 15% in 15–19 years old [1]. It is divided into a subclinical form which is only visible on ultrasound and a clinical form which can be recognized upon scrotal examination. The clinical form is further subdivided into three grades which are readily visible (grade 3), non-visible but palpable during normal examination (grade 2), and palpable during Valsalva maneuver only (grade 1). The condition is believed to arise due to compromised venous valves as the hydrostatic pressure increases during growth spurts. This means that varicoceles are most often found on the left side, as the left internal spermatic vein runs in a vertical course from the scrotum and into the left renal vein, whereas the right internal spermatic vein has a slanted course and drains into the inferior vena cava [2]. Another possible cause of left-sided varicoceles is increased pressure on this side due to compression of the internal spermatic vein between the superior mesenteric artery and the aorta [2]. Varicoceles may result in an uncomfortable feeling of pressure or scrotal pain, especially in relation to physical activity. In addition, the condition may compromise testicular growth on the affected side and it is associated with male infertility through unknown mechanisms [2]. For these reasons, varicocele treatment may be offered either by surgery or percutaneous radiological approaches. Generally, a subinguinal microsurgical approach with ligation of individual veins in the pampiniform plexus has been recommended as this has the lowest reported recurrence and complication rates, because it achieves closure of the veins distally to any collaterals while preserving testicular arterial supply and lymphatics [3]. Meanwhile, robot-assisted laparoscopic surgery offers both magnification and the possibility of increased surgical precision making it a possible alternative to microsurgery [4]. Therefore, robot-assisted laparoscopic ligation of the varicose veins has been introduced as a treatment for varicoceles at the Department of Pediatric Surgery, Rigshospitalet, Copenhagen, Denmark. The purpose of this paper is to present the initial experience, including results, complication rate, and surgical time.

## Materials and methods

The first robot-assisted laparoscopic varicocelectomy was performed in February 2016. We retrospectively reviewed all cases performed through September 2020. Information on patient characteristics, indications for surgery, surgical time, complication rates, and recurrences were collected from the electronic patient records. All varicoceles were diagnosed and graded with the patients standing in the upright position.

All surgeries were performed in general anesthesia with the Da Vinci Robotic SI system by the same experienced robotic surgeon. During the surgeries, one-to-four veins were ligated at the internal ring of the inguinal canal, while the testicular artery and lymphatics were spared. Following surgery, the patients received standard instructions to avoid strenuous exercise for 4 weeks and a clinical follow-up visit was scheduled 6 months after surgery. Neither semen parameters nor reproductive hormones were investigated, since we were dealing with an adolescent population.

Descriptive statistics were performed for all outcomes. Due to a homogeneous patient population and as the surgical procedure was standardized, analyses of possible predictors of surgical outcomes were not performed. The study was approved by the hospital as a quality assurance study without the need for further approvals in accordance with Danish law.

## Results

Forty-nine consecutive robot-assisted laparoscopic varicocelectomies were performed during the study period. The median patient age was 14 (range 10—17). All follow-up visits were performed between 6 and 7 months after surgery. One patient had Wolff–Parkinson–White syndrome and received a beta-blocker. Aside from this, no significant co-morbidities were noted. Two boys had previously undergone scrotal surgery; one had a left-sided appendices testis removed and the other had undergone bilateral fixation due to suspicion of sub-torsion. Finally, three boys presented with concurrent inguinal hernias, which were operated at the same time as the varicocele correction. Forty-eight had left-sided varicoceles and one had a bilateral varicocele. Forty-five were grade 3, three were grade 2, and one was grade 1. Four of the left-sided grade 3 varicoceles were recurrences from previous surgeries, one with the open retroperitoneal approach, one with traditional laparoscopy, and two with subinguinal microsurgery. All patients were primarily referred and treated due to discomfort and/or scrotal pain. Using an orchidometer, it was noted that 20 patients exhibited a reduction of 20% or more in testicular size on the affected side compared to the unaffected side.

Excluding patients who underwent concurrent hernia repairs, the median operating time from skin incision to the end of the procedure was 48 min (range 31–89 min) and the median console time was 18 min (range 7–55 min). Aside for a notably longer console time during the first surgery of 55 min, there were no clear signs of a learning curve in this regard. Forty-seven patients experienced no post-operative problems and were discharged the same day. The two remaining patients experienced pain and problems urinating, respectively. These issues had resolved by the first post-operative day where both patients were discharged. At 6-month follow-up, eight recurrences (16%) were noted on clinical examination. Two of these were in patients who had also been operated for inguinal hernias. No hematomas, infections, or hydroceles were noted. Scrotal pain had subsided in all patients. Catch-up growth of the affected testicles was noted upon clinical examination in the 19/20 boys who underwent surgery due to reduced testicular size. The boy who did not experience catch-up growth had a recurrence of his left-sided grade 3 varicocele.

## Discussion

Theoretically, the robot-assisted laparoscopic varicocelectomy has an advantage over the traditional laparoscopic approach, because structures are magnified and because ligation of the veins can be performed with enhanced precision. Only one previous publication has reported on robot-assisted laparoscopic varicocelectomy [5]. In this study, four pediatric patients (mean age 15.3 years) were treated for left-sided varicoceles with ligation of the gonadal vein. The mean operative time was 112 min and neither complications nor recurrences were seen. Therefore, robotic varicocelectomy needs to be further assessed in clinical trials such as ours and compared to those of established treatment modalities. Other surgical techniques for varicocele repair include open retroperitoneal high ligation [6] and traditional retroperitoneal laparoscopic varicocelectomy [7] with ligation of the internal spermatic vein, inguinal varicocelectomy with ligation of both the internal and external spermatic veins [8], and inguinal or subinguinal microsurgical varicocelectomy with ligation of individual veins in the spermatic cord [9]. As an alternative, varicoceles can be treated by radiological techniques with either embolization or sclerotherapy [10], 11. There is a lack of high-quality trials comparing these techniques, but, generally, the open retroperitoneal high ligation has the highest recurrence and complication rates, while the best results are obtained with the microsurgical approach. Thus, a meta-analysis from 2009 including 4,473 men found that the open retroperitoneal high ligation had a recurrence rate of 14.97% and a risk of hydroceles of 8.24% [3]. Meanwhile, the recurrence and hydrocele rates with the laparoscopic surgery were about 4.3% and 2.84%, respectively. For the inguinal approach without microsurgery, 2.63% experienced recurrences and 7.3% developed hydroceles. Microsurgical varicocelectomy had a recurrence rate of 1.05% and a post-operative hydrocele incidence of only 0.44%. Radiological interventions had the lowest success rate with technical failure in 13.05% and recurrence in 12.07%. Meanwhile, it should be highlighted that the success rate of varicocele repair varies considerably between individual studies and that successful outcomes have been reported for each technique in individual case series. In that regard, the crucial factor seems to be the steps taken to identify and treat parallel duplications of the internal spermatic vein, including renal and lumbar collaterals, which have been shown in the majority of adolescents with varicoceles [12]. While achieving this, steps should also be taken to avoid damaging small arteries and lymphatics and as this may cause testicular damage and hydroceles, respectively. Theoretically, all these goals are achieved using the microsurgical technique, while newer publications have also indicated that excellent results can be achieved through modifications of the radiological interventions [13]. In addition to these traditional varicocele treatments, a few recent case series have described the use of a subinguinal robot-assisted technique. This is similar to the microsurgical approach, only the microscope is replaced by the robot which is introduced after the initial incision. In 2008, the first case series including 8 patients was published [14]. The average operating time was 71.1 min and no complications or recurrences were observed. The authors did not note differences between subinguinal the traditional microsurgical approach. A subsequent study from 2013 [15] described 181 microsurgical subinguinal varicocelectomies in 154 patients who underwent surgery either for infertility or scrotal pain. The median surgical duration per side was 20 min (range 10–80 min). Two recurrences or persistence of varicocele occurred, one patient developed a small post-operative hydrocele and two patients had post-operative scrotal hematomas. In line with this, a recent study reported their results from 258 robot-assisted subinguinal varicocelectomies in 140 infertile men [16]. Here, the mean operative time per side was 57 min. The authors found a complication rate of 3.5%, including 7 (2.7%) hematomas and 2 (0.8%) hydroceles. In addition, there was a recurrence rate of 9.6% as assessed by Doppler ultrasound.

Due to these previous findings, microsurgical varicocele repair is currently considered the gold standard in varicocele treatment. As described, the robotic approach could play a future role in centers where robotic expertise is available based on theoretical advantages. This possibility is enforced by our relatively short surgical time, the lack of complications for an experienced robotic surgeon and the universal resolution of symptoms in successful cases. Considering the uncertainty inferred by the relatively low number of patients, this must be considered comparable to results with the more established treatments where over 90% are reported to be pain free after successful surgical intervention [16]. The overnight admission of two patients in our study is not generally seen with other approaches for varicocele repair. The problems leading to this may be caused by the laparoscopic approach, pain from the surgery itself and/or be due to the anesthesia, but the low number and retrospective nature of our study makes it difficult to assess the reason. While scrotal discomfort is universally accepted as a treatment indication, the association between varicoceles and male infertility remains somewhat controversial. In this regard, varicoceles are present in about 45% of infertile men [17] compared to only about 15% in the general population 1. In addition, varicoceles have been associated with reductions in semen quality, sperm function, and reproductive hormones [18–20]. On the other hand, most men with varicoceles are able to father children and it is unclear how male fertility is affected by the dilated veins [21]. Proposed theories include oxidative stress, scrotal hyperthermia, hypoxia, reflux of renal and adrenal metabolites, hormonal imbalances, and the formation of antisperm antibodies, but none of these theories have been convincingly demonstrated [22]. In our series, neither semen parameters nor reproductive hormones were investigated. This is in line with the current recommendations, since the uncertainties mean that there is no known way to predict how a varicocele in adolescents may predict future fertility potential. In fact, only about 20% of adolescents with a varicocele experience infertility in adulthood [23]. However, a case can be made for investigating semen parameters on an individual basis even in a population such as ours as this may help serve as a further incentive for surgery in cases of reduced semen quality. The use of WHO normal ranges for adults might be used in this context as previous research has shown that sperm concentration and motility are not correlated with age in adolescents with varicoceles [24]. Meanwhile, reduced testicular size on the affected side is recognized as a reason to consider treatment in adolescents, because catch-up growth has been shown in some case series [25]. We saw this in all successfully treated patients in our series patients lending further merit to the method. However, we also noted recurrences in 8 patients corresponding to a rate of 16% on clinical examination. Although our results may be affected by a learning curve, this is obviously high compared to case series with other methods. Therefore, further surgical experience and/or refinement of the technique is needed before the robot is ready for prime time in this setting. In this regard, careful follow-up is needed, as the recurrence rate may also be inherent to the surgical method. Thus, as described above, selective vein ligation can miss proximal parallel duplications of the internal spermatic vein [12]. This issue has been elegantly mitigated in the previous studies by performing venography with intravenous contrast and methylene blue to identify and confirm the closure of such veins [26]. A similar step could potentially be added to the robot-assisted approach.

The main weakness of the study is the retrospective design. Further, the assessment of recurrence and testicular catch-up growth was based on clinical examination alone rather than scrotal ultrasound.

## Conclusions

Our study represents the largest case series on robot-assisted laparoscopic varicocelectomies published to date. The results suggest that the procedure is feasible and safe but with a relatively high recurrence rate. The recurrence rate may be reduced with further experience and refinement of the technique but through follow-up is required to document this in future treatment series.
